# *Clostridium butyricum* Supplement Can Ameliorate the Intestinal Barrier Roles in Broiler Chickens Experimentally Infected With *Clostridium perfringens*

**DOI:** 10.3389/fphys.2021.737481

**Published:** 2021-09-24

**Authors:** Xiao Xu, Shunli Yang, Joshua Seun Olajide, Zigang Qu, Zhenxing Gong, Jing Wang, Yanbing Zhang, Heng Wang, Ling Xiong, Kun Zhang, Enmin Zhou, Jianping Cai

**Affiliations:** ^1^State Key Laboratory of Veterinary Etiological Biology, Key Laboratory of Veterinary Parasitology of Gansu Province, Lanzhou Veterinary Research Institute, Chinese Academy of Agricultural Sciences, Lanzhou, China; ^2^Department of Preventive Veterinary Medicine, College of Veterinary Medicine, Northwest A&F University, Yangling, China; ^3^Jiangsu Co-innovation Center for the Prevention and Control of Important Animal Infectious Disease and Zoonoses, Yangzhou University, Yangzhou, China

**Keywords:** *Clostridium butyricum*, *Clostridium perfringens*, necrotic enteritis, gut health, intestinal barrier

## Abstract

Necrotic enteritis (NE), caused by *Clostridium perfringens*, is an economically important disease in the broiler. Among normal flora in the broiler intestinal region, *Clostridium butyricum* has been identified as a probiotic agent that reduces the susceptibility of broilers to *C. perfringens*. However, the effects of *C. butyricum* supplement on broiler intestinal integrity during NE are largely unknown. In this study, we investigated the effects of *C. butyricum* on the growth performance, intestinal morphology and barrier function, and the functions of immune-related cytokines under NE in broilers. Chickens were divided into five groups: control group (NC), supplement *C. butyricum* only group (CB), NE-infected group (PC), supplement *C. butyricum* from Day 14 (NECB1) to Day 22 NE-infected group, and supplement *C. butyricum* from Day 1 (NECB2) to Day 22 NE-infected group. The results showed that there were significantly decreased average daily weight gain and increased feed conversion rate in the infected group (PC) compared with the *C. butyricum*-supplemented groups (NECB1 and NECB2) through the diet. Histopathological observation on the Hematoxylin–Eosin staining avian small intestine sections revealed that supplementation of *C. butyricum* (NECB1 and NECB2) could increase the intestinal villus height/crypt depth and lessen the intestinal damage under NE. ELISA and Limulus test showed that broilers infected with NE (PC) had higher serum IgA and lipopolysaccharide content; however, after *C. butyricum* supplementation (NECB1 and NECB2), they returned to a normal level. Furthermore, real-time PCR and Western blot results indicated that compared with PC, supplementing *C. butyricum* (NECB1 and NECB2) could initialize the expressions of genes related to the intestinal barrier-associated molecules (such as CLDN-1, CLDN-3, OCLN, MUC2, ZO-1, and CLDN5), cytokines (such as IL-10, IL-6, and TGFB1), and *C. perfringens plc* gene expression. Moreover, the results detected by the Ussing chamber suggested that *C. butyricum* (NECB1 and NECB2) could amend the decrease in conductivity value and short-circuit current value caused by NE. In addition, NECB2 significantly reduced the upregulation of fluorescein isothiocyanate–dextran flux caused by the NE disease. In conclusion, these findings suggest that dietary supplementation of *C. butyricum* in broilers with NE improved chicken growth performance, intestinal integrity and barrier function, and immunological status. Notably, no statistical difference was observed with the addition of *C. butyricum* on day 1 or day 14.

## Introduction

Necrotic enteritis (NE) in broilers is caused by the strains of *Clostridium perfringens* types A, C, and G (Opengart and Boulianne, 2019) and is sometimes accompanied by co-infection with *Eimeria maxima* (Opengart and Songer, 2013). Clinically, broiler NE caused by *C. perfringens* is mainly characterized by mucosal necrosis of the small intestine. After the onset of the disease, the intestinal wall becomes thin and brittle, resulting in intestinal bleeding. In some severe cases, the intestinal cavity is filled with blood. Acute cases of NE in broilers are marked with diarrhea, anorexia, messy feathers, depression, bloody stools, and coal tar feces, resulting in yolk pedicle contraction, intestinal fibrosis, and necrosis. Broilers with NE may also develop the subclinical disease, with mild or no symptoms of diarrhea, and gradually progress to chronic wasting (Gholamiandehkordi et al., 2007; Timbermont et al., 2011). Although the mortality caused by NE in broilers with subclinical symptoms is not high, the NE in broilers results in severe economic losses for the poultry industry. This disease not only increases the mortality of the flock but also reduces the feed conversion rate directly caused by the severe damage to the intestines of broilers, delays the time to slaughter, and drives over 6 billion dollar losses in the poultry industry worldwide (Moore, 2016).

The use of antibacterial growth agents and anticoccidial drugs in the feed is essential in controlling poultry diseases. However, the widespread use of antimicrobials has also led to bacterial resistance and subsequently to restrictions or prohibitions on antibiotics in several countries around the world. Since the European Union started to ban antibiotic growth promoters in 2006 completely (EU, 2003), the incidence and prevalence of NE have increased significantly (Van Immerseel et al., 2009). Moreover, coccidiosis/coccidiasis, one of the main predisposing factors of NE, caused the incidence of *C. perfringens*-associated enteritis to increase yearly (Williams, 2005). Hence, alternatives to antibiotics, such as probiotics, have received significant attention in improving the broiler industry.

Probiotics can be used as growth promoters and, in some cases, can be used to control intestinal pathogens (Zhou et al., 2020). Moreover, probiotics can maintain normal intestinal function and regulate innate and adaptive immunity, and the epithelial barrier function (Choct, 2009; Viswanathan et al., 2009; Gaggìa et al., 2010).

Some probiotics have beneficial effects in broilers with NE, such as *Lactobacillus acidophilus* (Li et al., 2018) and *Bacillus coagulans* (Wu et al., 2018). Among the available probiotics, *C. butyricum*, a gram-positive anaerobic rod isolated from the intestines of healthy humans and animals, has many functions in animal breeding, such as regulating animal intestinal health (Liao et al., 2015). *C. butyricum* promotes the growth performance and immune function of broilers, and it is beneficial to balance the intestinal microbiota in broilers (Liao et al., 2015). Zhang et al. (2016) showed that the dietary supplementation of *C. butyricum* is beneficial to the maintenance of the intestinal barrier. *C. butyricum* also has a beneficial effect on broilers infected by NE (Huang et al., 2019). Some studies have also demonstrated that *C. butyricum* can induce short-chain fatty acids such as lactic acid and butyric acid in the digestive tract (Nakanishi et al., 2003). Moreover, the butyric acid produced by *C. butyricum* can increase the growth of beneficial bacteria and inhibit pathogenic bacteria, such as *C. perfringens* (Kong et al., 2011). Notably, the butyric acid also plays a vital role in the nutritional properties of epithelial cells and the inhibitory effect on pathogens in the intestine (Meimandipour et al., 2010); furthermore, it contributes to epithelial cell development in the intestine (Leeson et al., 2005). Butyric acid can also affect tight junction (TJ) expression and epithelial cell proliferation to maintain the structure of villus and intestinal integrity (Timbermont et al., 2010; Eeckhaut et al., 2011).

*C. butyricum* is a potent antibiotic substitute and an effective probiotic in livestock and poultry breeding. However, there are few reports on the effect of *C. butyricum* in broilers with *C. perfringens*-induced NE. Therefore, we evaluated the biological effects of *C. butyricum* on *C. perfringens*-infected broilers in terms of growth performance, secretion of serum IgA and lipopolysaccharide (LPS), intestinal barrier function, immune-related gene expression, and Fluorescein isothiocyanate–dextran (FD4) flux.

## Materials and Methods

### Chickens, Diet, and Experimental Design

Coccidia- and *C. perfringens*-free 1-day-old Ling-nan yellow-feathered broilers (Xinxisheng Biotechnology, China) were raised in the animal house of the Lanzhou Institute of Veterinary Medicine, Chinese Academy of Agricultural Sciences (LVRI, CAAS). *E. maxima* (GD strain) and *C. perfringens* strain toxinotype G (Hu et al., 2015) were isolated and maintained by the laboratory for swine/poultry intestinal infections and mucosal immunity of the LVRI, CAAS. *C. butyricum* (GCMCC0313.1) was acquired from Baifude Biotechnology (Wuxi, China). The feed used in this experiment was customized from Xinxiwang Feedstuff Company (Lanzhou, China) without any anticoccidial and growth-promotion antibiotic additives (Table 1). The animal cages in one house were divided into five groups, each with 10 broilers, including the negative control group (NC), *C. butyricum* group (CB), NE infection (PC), and two groups of NE broilers supplemented with *C. butyricum* from day 14 (NECB1), and from day 1 (NECB2). In addition, two groups (*E. max* and ECB) were set up with or without *C. butyricum* from day 1 and were administered with 3 ×10^4^ *E. maxima* sporulated oocysts to exclude the effect of *C. butyricum* on *E. maxima* infection (Table 2).

**Table 1:** Composition of basal diet.

**Composition**	**Content (g/kg)**
Corn	612.4449
Soybean meal 43	190.0000
Cotton meal 50	70.0000
Corn gluten meal 60	55.0000
Bran	25.0000
Dicalcium phosphate	17.0000
Stone powder	12.0000
Soybean Oil	5.0000
L-Lysine sulfate (70%)	4.4000
Sodium chloride	3.0000
999 Meat and poultry trace element premix	2.0000
DL-Methionine (98.5%)	1.4000
Choline chloride (60%)	1.0000
L-Threonine (98.5%)	0.7000
25%Tryptophan	0.5000
RJ-dv1301 Poultry, rare birds for livelihood	0.3500
Enramycin premix (8%)	0.1250
Homotropin (mannanase)	0.0800
Flavomycin premix (8%)	
Chlortetracycline premix (25%)	
	1,000.000

**Table 2:** Experimental design.

**Group**	***Clostridium butyricum***	**Infectious dose**
NC	/	/
CB	2 × 10^8^ CFU/g (D1-D22)	/
PC	/	3 × 10^4^ *Eimeria maxima* and 5 × 10^8^ CFU/mL *Clostridium perfringens*
NECB1	2 × 10^8^ CFU/g (D14-D22)	3 × 10^4^ *E. maxima* and 5 × 10^8^ CFU/mL *C. perfringens*
NECB2	2 × 10^8^ CFU/g (D1-D22)	3 × 10^4^ *E. maxima* and 5 × 10^8^ CFU/mL *C. perfringens*
*E. max*	/	3 × 10^4^ *E. maxima*
ECB	2 × 10^8^ CFU/g (D1-D22)	3 × 10^4^ *E. maxima*

*NC, control group; CB, C. butyricum only group; PC, NE-infected group; NECB1, supplement C. butyricum from Day 14 to Day 22 NE-infected group; NECB2, supplement C. butyricum from Day 1 (NECB2) to Day 22 NE-infected group*.

For predisposing the pathogenesis of NE caused by *C. perfringens* infection, the stored *E. maxima* oocysts at 4°C were passaged using 14 day-old coccidia-free broiler chickens. The progeny oocysts of *E. maxima* were purified, sporulated, and counted according to standard approaches (Conway and McKenzie, 2007). For inoculating *E. maxima* oocysts through the crop infusion of the broiler to predispose the infection of *C. perfringens*, these purified oocysts should be stored at 4°C for up to 20 days.

Type G *C. perfringens* strain stored at −80°C was resuscitated in fluid thioglycollate medium (with 0.5% D-cycloserine) under an anaerobic culture system (Whitley DG250, UK, gas conditions as 80% N_2_, 10% H_2_, 10% CO_2_) at 37°C for 18 h. Then, the concentration of *C. perfringens* was adjusted to 5 × 10^8^ CFU/mL using McFarland nephelometer (HuanKai Biotechnology Company, Guangdong, China) (Bollela et al., 1999) and real-time recombinase polymerase amplification (Xu et al., 2019).

The recommended dosage of *C. butyricum* powder for broiler chickens is 200–400 g/t compound feed. Before inoculation, we counted the living organisms in this product with the reference method in Veterinary Pharmacopeia of the People's Republic of China, to ensure enough designed dosage. The *C. butyricum* powder was directly mixed with feed in the recommended dosage (2 × 10^8^ CFU/g) by a mechanical mixer and then put into the manger for eating by chickens *ad libitum* (Table 2). The infected groups (PC, NECB1, and NECB2) were orally administrated with 3 × 10^4^ *E. maxima* sporulated oocysts in 1 mL of suspension on day 14 and orally inoculated three times a day for 3 days with 5 × 10^8^ CFU/mL of *C. perfringens* on days 18, 19, and 20 (Table 2) (Xu et al., 2018). The uninfected groups (NC and CB) were administrated with the same amount of sterile saline at the corresponding times.

### Ethics Statement

All animal experiments and experimental procedures were approved by the State Key Laboratory of Veterinary Etiological Biology, Lanzhou Veterinary Research Institute, Chinese Academy of Agricultural Sciences, China.

### Growth Performance and Sample Collection

The body weight and feed consumption of the broilers were recorded on days 1, 13, and 22 for evaluating the average daily weight gain (ADG), average daily feed intake (ADFI), and feed conversion ratio (FCR) on days 13 and 22. The chickens were sacrificed by cervical dislocation on day 22, and then, blood samples were taken from the heart at once. The collected blood samples were incubated at 37°C for 1 h and then at 4°C for 10 h. Serum was isolated by centrifugation at 8,000 rpm for 10 min and stored at −80°C. The abdominal cavity was opened, and the small intestine lesions were examined. Then, the isolated intestine was cut longitudinally and photographed, and the intestinal contents were taken (quickly frozen in liquid nitrogen and stored at −80°C) for *C. perfringens* enumeration. The small intestine tissues in front of Meckel's diverticulum were cut into 2 cm in length, quickly frozen in liquid nitrogen, and then stored at −80°C for RNA and protein extraction. Additional pieces in the length of 1 cm were stored in 4% paraformaldehyde at 4°C for pathohistological examination. Moreover, the 1 cm length of small intestine tissue post the Meckel's diverticulum was taken and immediately used for Ussing chamber testing.

### Intestinal Histomorphology

The small intestine samples fixed with 4% paraformaldehyde were then dehydrated, paraffin-embedded, sectioned to 4 μM using Leica DM2016 microtome (Wuhan Junjie Electronics, China), and fixed on the glass slide. The slices were stained with hematoxylin–eosin (HE, Servicebio, China) and then scanned with a panoramic slice scanner (3DHistech, Hungary). The height of the villus and the depth of crypts were measured.

### Serum Immunoglobulin a Assay

The serum immunoglobulin A (IgA) was determined using a broiler immunoassay A ELISA (Cusabio, China). In brief, the references for the calibration curve and samples were added to every microplate at the same time, and each reference and samples have by triplicate biological replicates. Then, the absorbance was measured at 450 nm wavelength with a microplate reader (SpectraMax M5, Molecular Devices, United States) within 5 min after the reaction was terminated. After obtaining the absorbance value, a standard curve was generated to calculate the IgA concentration fold-change.

### Serum LPS Assay

The serum LPS in broilers was detected using the Limulus test (Iwanaga, 2007) and with the LPS detection Tachypleus Amebocyte Lysate kit (Xiamen Bioendo Technology, China) following the kit manual. The absorbance value was measured at 405 nm, and a standard curve was generated to calculate the LPS concentration.

### *E. maxima* Oocyst Output

To determine the number of *E. maxima* oocysts per **gram** of excreta, 10 g of fresh feces was collected from the four corners and the middle of each cage of *E. max* and ECB groups on day 7 after *E. maxima* infection. Briefly, 10 g of feces was soaked in 10 mL of tap water for 24 h at 4°C in a 50 mL beaker that is tightly covered with a lid, and then, the oocysts were counted according to a standard procedure (Conway and McKenzie, 2007).

### Intestinal *C. perfringens* Enumeration

The genomic DNA of broiler gut microbiota was extracted using the stool genome extraction kit (Mobio12888, Qiagen, Germany). The obtained DNA was used as the template, and bacterial 16s rDNA was used as the house-keeping gene for detecting *C. perfringens* alpha-toxin gene, *plc*, expression (Table 3) with the quantitative PCR method (Nagpal et al., 2015). For *plc* quantitation, 10 μL of ChamQ SYBR qPCR Master Mix (Q311-02, Vazyme, China), 1 μL of forward primer, 1 μL of reverse primer, 2 μL of DNA, and 6 μL of ddH_2_O were added into one PCR tube. The amplification reaction was run according to the program of pre-denaturation at 95°C for 30 s, denaturation at 95°C for 10 s, annealing at 59°C for 30 s, and melting curve after 40 cycles on CFX96TM Real-time fluorescence quantitative PCR instrument (Bio-Rad, United States). The Ct value 2^−ΔΔCt^ was calculated to analyze the relative expression difference.

**Table 3:** Quantitative real-time PCR primer sequences.

**Gene**	**Forward primer**	**Reverse primer**	**Genbank accession number**
GAPDH	CACGCCATCACTATCTTC	GACTCCACAACATACTCAG	NM_204305.1
CLDN-1	GTGTGTTTGTTGCTGTGA	ACTCTGTTGCCATACCAT	NM_001013611.2
CLDN-3	GTCATCTTCCTGCTCTCC	AGCGGGTTGTAGAAATCC	NM_204202.1
CLDN-5	ACCATCTACATCCTCTGC	GTCGTAGAAGTCGCTGAT	NM_204201.1
OCLN	CCAGCGGTTACTACTACA	CAGGATGACGATGAGGAA	NM_205128.1
MUC-2	TTACCACCATAGTTACCACAA	CACTCAGACCAATCACAGA	NM_001318434.1
ZO-1	ATGAATGAAGGATGGTATGG	GATGTATGTCTGCTGTCTG	XM_015278981.2
IL-6	CTCCTCGCCAATCTGAAG	CTCACGGTCTTCTCCATAA	NM_204628.1
IL-10	GCCATCAAGCAGATCAAG	CTTCCTCCTCCTCATCAG	NM_001004414.2
TGFB1	CGGATGAGAAGAACTGCT	CCTTTGGGTTCGTGGATC	NM_001318456.1
TNF-α	AGCCTATGCCAACAAGTA	GGTCATAGAACAGCACTAC	NM_204267.1
16s rDNA	GTGCTACAATGGCTGGTA	CTACAATCCGAACTGAGACT	NR_121697.2
*plc*	AGTCTACGCTTGGGATGGAA	GTGATTCCCCTGTGTCAGGT	AY823400.1

### RNA Extraction, cDNA Synthesis, and Real-Time PCR

RNA extraction was performed using TRIzol (ThermoFisher, United States) following the instructions of the manufacturer. Total RNA concentration was measured using NanoPhotometer NP80 (Implen, Germany) and reverse transcribed to cDNA using the Prime Script RT reagent kit (RR047A, Takara, Japan) as described in the manual. The obtained cDNA was stored in a freezer at −20°C.

Primers for real-time PCR designed by Beacon Designer 8 are shown in Table 3. The fluorescence quantitative kit instructions were followed to do this experiment (RR820A, Takara, Japan). The real-time PCR reaction tube contained 1 μL of forward or reverse primers, 1 μL of template DNA, 10 μL of TB Green enzyme, and water, with a total volume of 20 μL, and the mixture and the samples were thoroughly mixed. The reaction program was pre-denaturation at 95°C for 5 min, denaturation at 95°C for 30 s, and annealing at 59°C for 30 s. Forty cycles were performed, and the melting curve was inserted. The Ct value obtained from 2^−ΔΔCt^ was used to analyze the relative expression difference.

### Western Blot

Total protein was extracted according to the procedure for RIPA lysis buffer (P0013B, Beyotime, China). The total protein concentration was measured using the BCA kit (T9300A, Taraka, Japan). Protein was resolved on 12% SDS–PAGE gel and transferred to a polyvinylidene difluoride membrane (Merck Millipore, Germany). Skimmed milk powder (5%) at room temperature was used to seal the membrane and then incubated with the primary antibody consisting of GAPDH 1:10,000 (Proteintech, United States), CLDN-1 1:125 (ThermoFisher, United States), OCLN 1:125 (ThermoFisher, United States), and *C. perfringens plc* 1:500 (Bioss, China) overnight at 4°C. This process was followed by incubation using the secondary antibody (Qiyan Biotech, China) for 1 h at room temperature. After exposure to the electrochemiluminescence kit (Merck Millipore, Germany), the film obtained was scanned for protein band and analyzed using the ImageJ software.

### Ussing Chamber Testing

With the salt bridge in place, the sample holder was empty as required, and 5 mL of Krebs–Henselet (K–H) solution containing 117 mM NaCl, 4.7 mM KCl, 1.2 mM MgSO_4_·7H_2_O, 1.2 mM KH_2_PO_4_, 2.418 mM NaHCO_3_, 11.1 mM GluCose, and 2.56 mM CaCl_2_ was added to each chamber, with the passage of oxygen to set the resistance and voltage to zero and turned on the VCC MC6 (Physiologic Instruments, United States). After debugging the instrument, the K–H solution was eliminated, the sample holder was removed, and then ~1 cm of the intestinal tissue was immediately supplied with oxygen (95% O_2_ and 5% CO_2_) at 4°C and pre-cooled with K–H solution after the longitudinal cut. The tissue was fixed in a 0.3 cm^3^ sample holder and filled with 5 mL of K–H solution, and the sample holder was reinstalled. The alternative values of short-circuit current value (Isc) and conductivity value (Gt) were recorded for 20 min, and when the curve was in a stable state, 100 μL of FD4 (0.375 mg/mL) solution was added to the mucosal side, while 100 μL of liquid was aspirated from the serosal side after 30 min (Hu et al., 2013). And a microplate reader was used to record the value of the excitation wavelength at 490 nm and emission wavelength at 520 nm.

### Statistical Analysis

Average daily weight gain, feed consumption, villus height, crypt depth, and Ussing chamber testing data used the AOV function in the R package to perform two-way ANOVA and calculated the *p* value. Tukey multiple comparisons and graph drawing were run on GraphPad Prism (v 7.0). One-way ANOVA was used to analyze the differences between different treatment groups and group NC in the remaining data, and graphs were generated using the GraphPad Prism.

## Results

### Growth Performance

**Figure 1:**
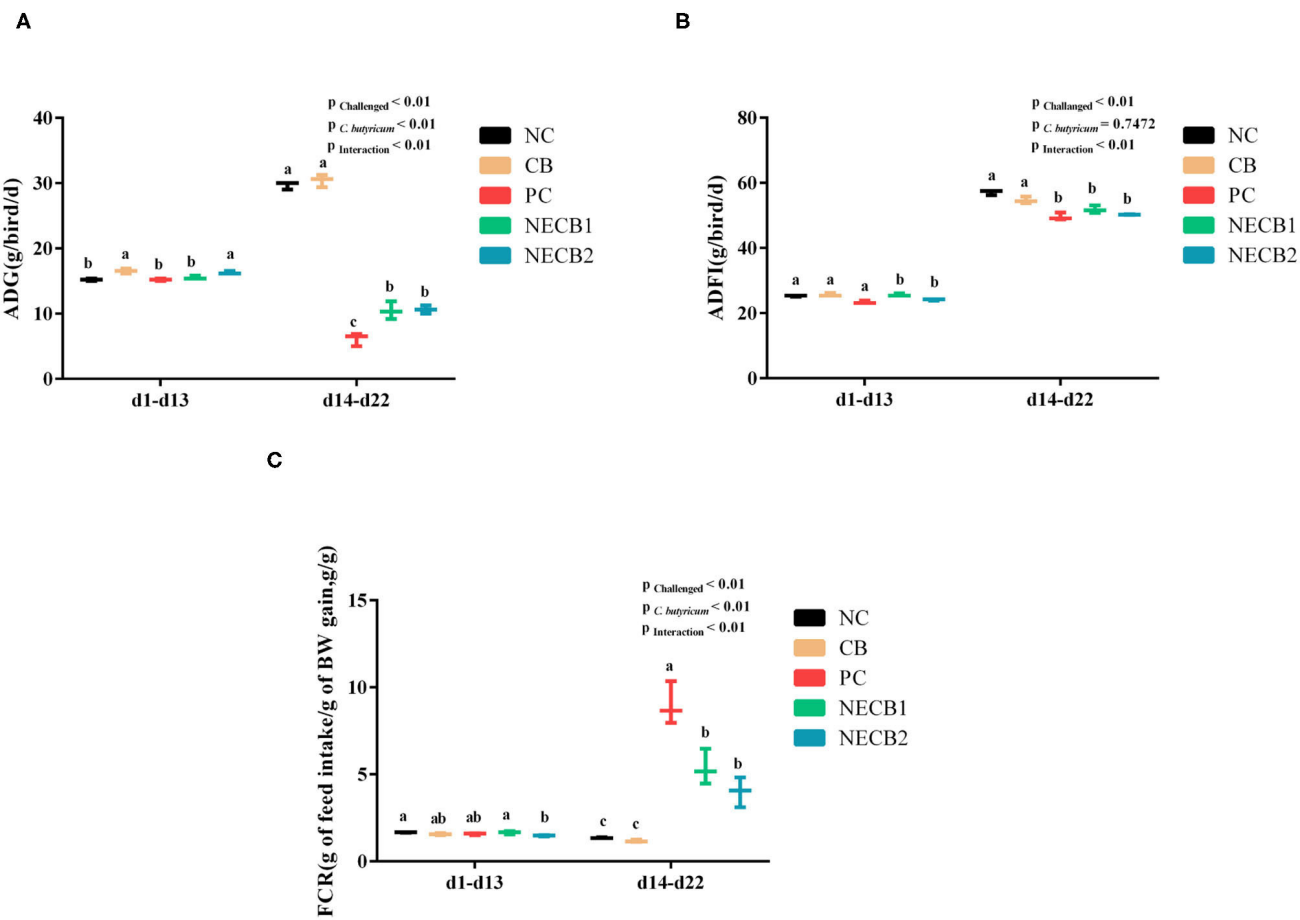
Effect of diet *Clostridium butyricum* supplementation on ADG, ADFI, and FCR of broilers infected with necrotic enteritis. **(A)** ADG: average daily weight gain. **(B)** ADFI: average daily feed intake. **(C)** FCR: feed conversion rate. Each result represents the mean ± SEM (*n* = 3). The different lowercase letters on the figure indicate significant differences (*p* < 0.05).

The growth performance of broilers was evaluated by the analysis of ADG, ADFI, and FCR values. At 14–22 days, the decrease of ADG and ADFI caused by NE was lessened by the supplementation of *C. butyricum* (NECB1 and NECB2) (Figures 1A,B). The increase of FCR in NE broilers was significantly reduced (*p* < 0.01) at 14–22 days in groups NECB1 and NECB2 compared with the PC group (Figure 1C).

### Pathohistological Examination

**Figure 2:**
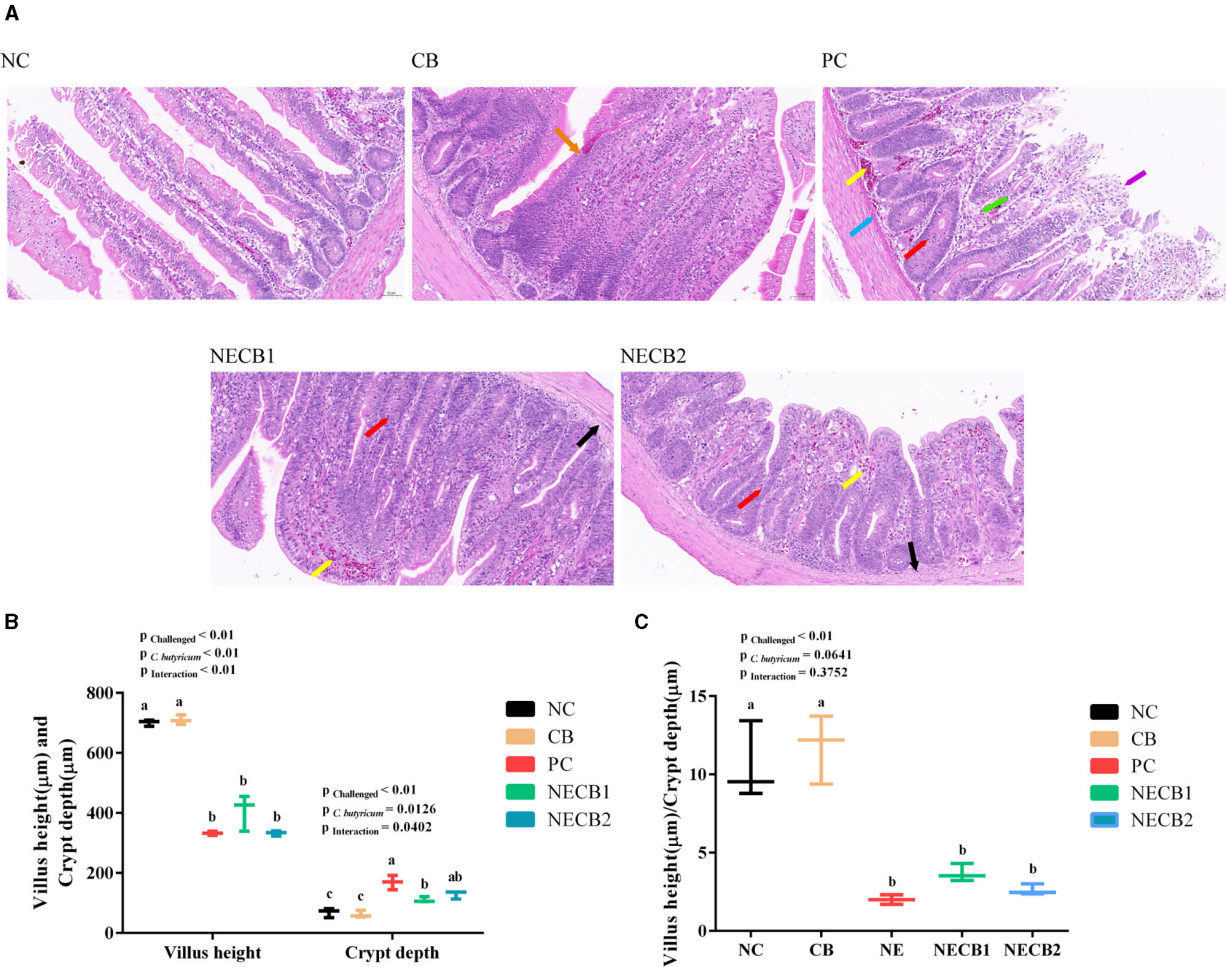
Effect of diet *C. butyricum* on the pathological section results, villus height, and crypt depth of necrotic enteritis-infected broilers. **(A)** Pathological section results. Yellow arrow: capillary congestion; red arrow: inflammatory cell infiltration; black arrow: swelling of mucosal muscularis cells, the cytoplasm is loose and lightly stained or vacuolated; blue arrow: eosinophils; orange arrow: intestinal villi merge; purple arrow: exposed lamina propria; and green arrow: increased diffuse lymphoid tissue. Scale bar=50 μm. **(B)** Villus height and crypt depth. **(C)** Villus height/crypt depth ratio. Each result represents the mean ± SEM (*n* = 3). The different lowercase letters on the figure indicate significant differences (*p* < 0.05).

The intestinal macroscopic lesions are shown in Supplementary Figure 1. Compared with NC, the intestinal wall in the PC became thinner and brittle and had obvious bleeding points. The pathohistological visualizations are shown in Figure 2A. From that, we could find that the structure of the mucosal layer of the small intestine tissue was intact, and the intestinal cell or intestinal glands were abundant and arranged tightly in group NC. On the contrary, the villi in the intestinal epithelial cells fell off, and the lamina propria was exposed in PC. Furthermore, lamina propria capillaries (yellow arrow) were with mild congestion, and inflammatory cell infiltration was raised in the intestinal gland epithelium (red arrow) with increased diffuse lymphoid tissue (green arrow) and eosinophilic cytoplasm (blue arrow) in PC. However, in group NECB1, and similarly in group NECB2, although the intestinal villi shortened, more intestinal villi fused and a small amount of inflammatory cell infiltration in the intestinal gland epithelium was observed (red arrow).

The villus height, crypt depth, and their ratios were analyzed with Caseviewer software (Figures 2B,C), and it was observed that NE infection (PC) had a significant impact on the intestinal villus height and crypt depth (*p* < 0.01, *p* < 0.05). Compared with NC, NE infection (PC) could decrease significantly the villus height and increased significantly the crypt depth. However, *C. butyricum* supplement (NECB1 and NECB2) could reduce the decrease of villus height and increase of crypt depth caused by NE infection (PC). The villus height/crypt depth ratio (Figure 2C) revealed a significant interaction (*p* < 0.05) between the NE infection and the *C. butyricum* supplementation. However, it is notable that although supplementing *C. butyricum* (NECB1 and NECB2) could depress the declination of villus height/crypt depth ratio caused by NE infection (PC), no significant difference between NECB1 and/or NECB2 with PC was observed.

In the *E. max* group, a large number of shed mucosal epithelial cells in the intestinal lumen and eosinophils infiltrating (blue arrows) into the cavities of the intestinal villi were found. Moreover, a moderate inflammatory cell infiltration could also be detected in the mucosal epithelium and intestinal gland epithelium (red arrow). But in group ECB, there was only a moderate amount of inflammatory cell infiltration (red arrow) and little loss of mucosal epithelium at the top of the villi (Supplementary Figure 2B).

### Serum IgA and LPS Content

**Figure 3:**
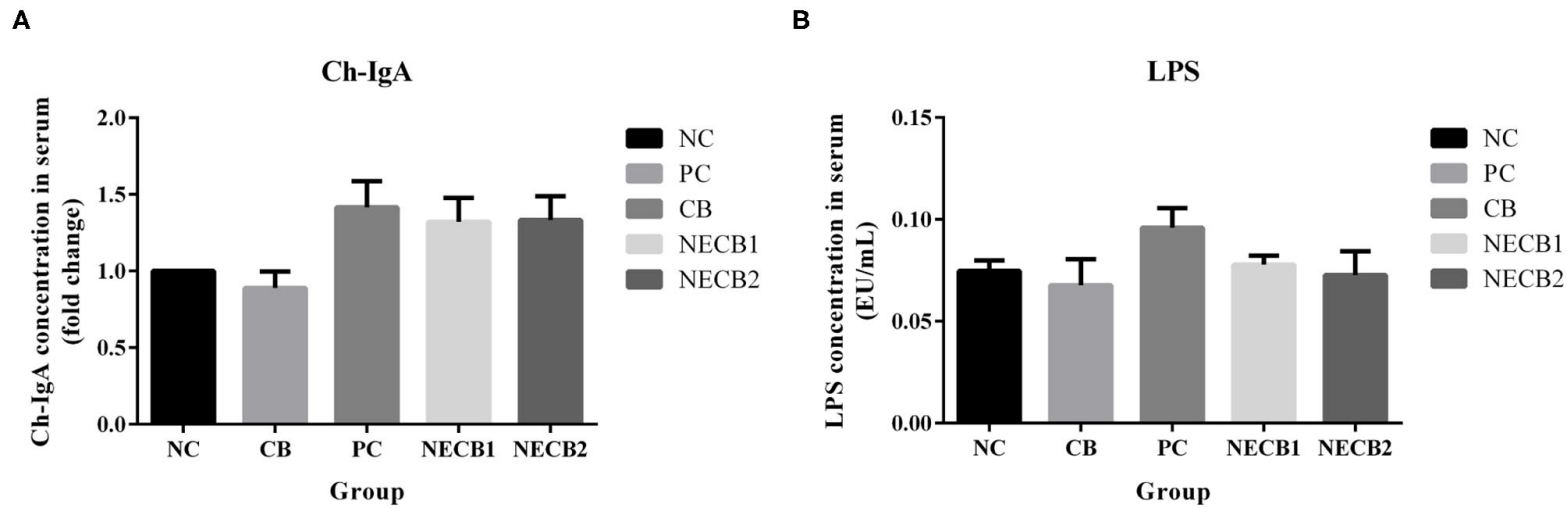
Effect of diet *C. butyricum* on serum immunoglobulin A (IgA) and lipopolysaccharide (LPS) in broilers infected with necrotic enteritis. **(A)** IgA. **(B)** LPS. Each result represents the mean ± SEM (*n* = 3).

Figures 3A,B presents the variations of serum IgA and LPS levels between the NC and the trial groups. In comparison with NC, although serum IgA was elevated in PC, this dynamic trend could be ameliorated by supplementing *C. butyricum* (NECB1 and NECB2), and there were no significant differences among them. Similarly, *C. butyricum* supplementation did not significantly lower the increase of serum LPS levels in PC.

### *E. maxima* Oocyst Count

As compared with *E. max*, adding *C. butyricum* in the diet had no significant impacts on the *E. maxima* oocyst shedding in ECB (Supplementary Figure 3).

### Intestinal Barrier Function-Related Genes, *C. perfringens plc* Gene, and Protein Expression

**Figure 4:**
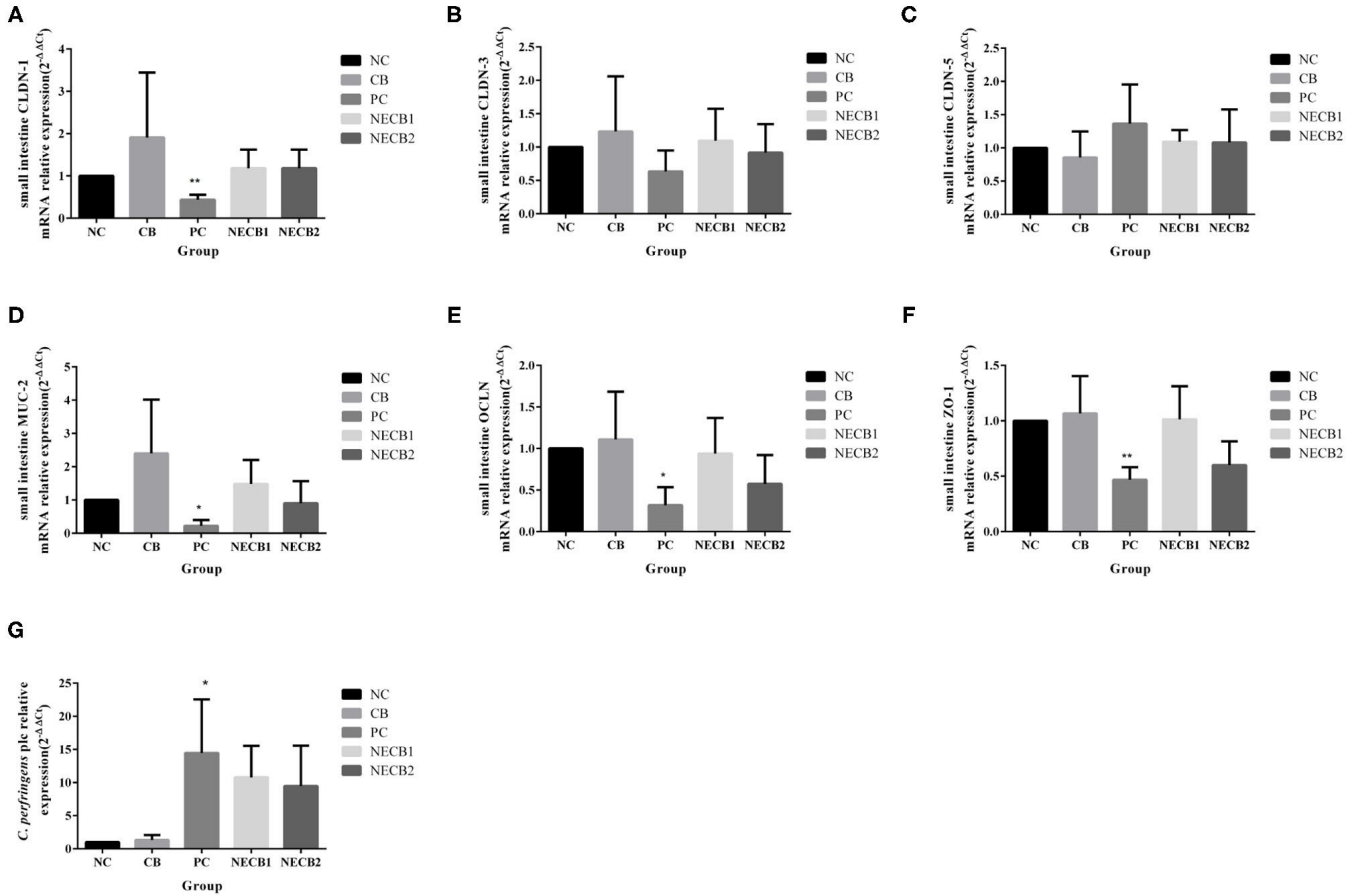
Relative mRNA expression intestinal barrier function-related in the small intestine and *Clostridium perfringens plc* gene in small intestine contents. **(A)** CLDN-1. **(B)** CLDN-3. **(C)** CLDN-5. **(D)** MUC-2. **(E)** OCLN. **(F)** ZO-1. **(G)** CPplc. Each result represents the mean ± SEM (*n* = 3). ^*^Represent *p* < 0.05, ^**^represent *p* < 0.01.

**Figure 5:**
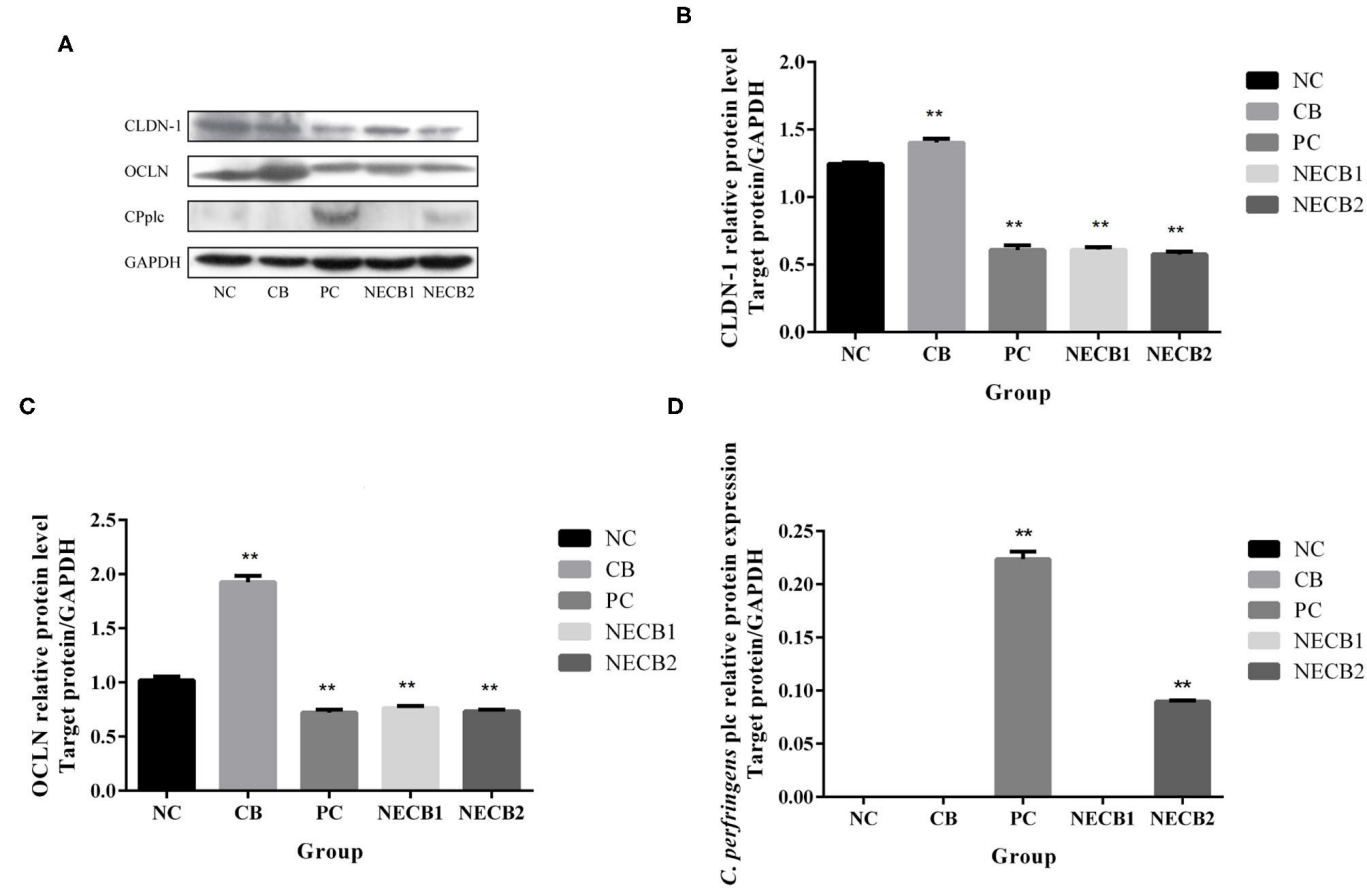
Protein expression in the small intestine. **(A)** Western blot analysis results of GAPDH, CLND-1, OCLN, CPplc. **(B)** CLDN-1. **(C)** OCLN. **(D)** CPplc. Each result represents the mean ± SEM (*n* = 3). ^**^Represent *p* < 0.01.

Necrotic enteritis infection resulted in the drop of TJ proteins, such as CLDN-1, CLDN-3, OCLN, and ZO-1, and mucosal barrier-associated protein MUC-2 mRNA expression; especially a significant fall in MUC-2, OCLN (*p* < 0.05), CLDN-1, and ZO-1 (*p* < 0.01) was observed (Figures 4A,B,D–F). However, elevatory CLDN-5 and significantly increased *plc* expression (*p* < 0.05) has been revealed in PC (Figures 4C,G). On the other hand, addition of *C. butyricum* (NECB1 and NECB2) retarded the decrease of CLDN-1, CLDN-3, MUC-2, OCLN, and ZO-1 caused by NE infection, although no significant differences were observed among them. Western blot results (Figure 5) showed that in PC, the expression of CLDN-1 and OCLN significantly decreased (*p* < 0.01) and increased the expression of *plc*. The addition of *C. butyricum* (CB) significantly increased the expression of CLDN-1 and OCLN contrasted with NC. Also, the addition of *C. butyricum* in NECB1 and NECB2 lightened the expression *plc* caused by NE.

### Immune-Related Cytokines and Growth-Related Factor Gene Expression

**Figure 6:**
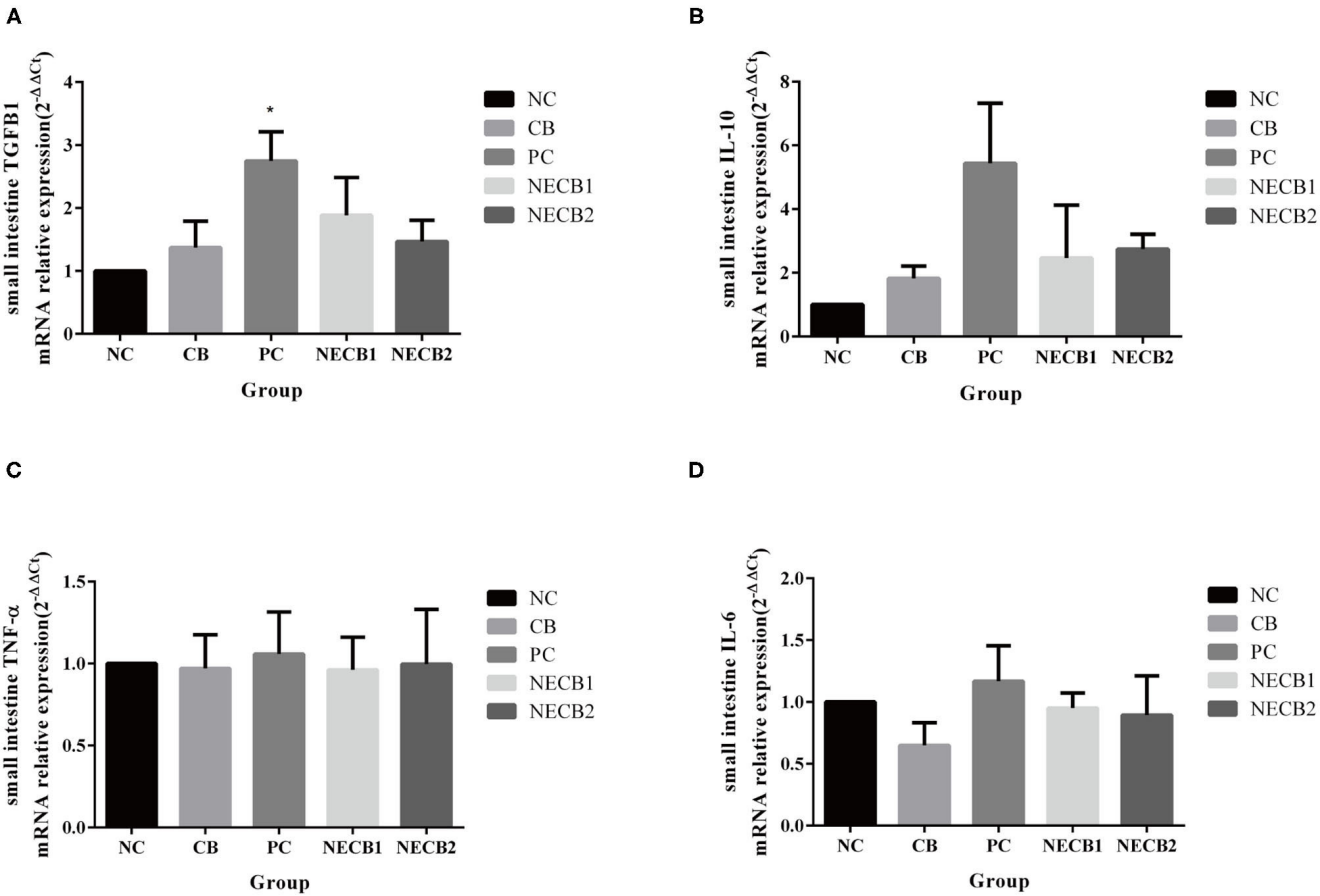
Effect of diet *C. butyricum* on the expression of cytokines related-genes in the small intestine of broilers infected with NE. **(A)** TGFB1. **(B)** IL-10. **(C)** TNF-α. **(D)** IL-6. Each result represents the mean ± SEM (*n* = 3). ^*^Represent *p* < 0.05.

Necrotic enteritis infection (PC) increased TGFB1 and immune-related factors, such as IL-10 and IL-6, compared with NC. However, supplementation of *C. butyricum* (NECB1 and NECB2) alleviated the upregulation of TGFB1, IL-10, and IL-6 in PC (Figure 6).

### Data From Ussing Chamber Testing

**Figure 7:**
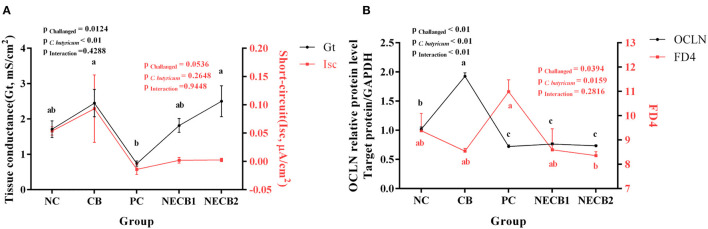
Ussing chamber experiment results. **(A)** Changes in short-circuit current (Isc) and conductance (Gt). **(B)** OCLN expression level and FD4 flux. Each result represents the mean ± SEM (*n* = 3). The different lowercase letters on the lines indicate significant differences (*p* < 0.05).

In comparison with the NC group, there was no significant decrease of Gt in the PC group. The addition of *C. butyricum* prevented the decrease in Gt caused by NE, especially in NECB2, where Gt significantly increased (*p* < 0.01). The Isc of PC also decreased, and supplement *C. butyricum* (NECB1 and NECB2) could relieve the decrease of Isc caused by NE. In this case, no statistical difference was observed (NECB1 and NECB2, Figure 7A). Furthermore, the FD4 flux in PC increased, while *C. butyricum* addition in NECB2 could significantly inhibit the increase of FD4 flux caused by NE infection (*p* < 0.01). The fluorescence intensity of FD4 and the expression level of OCLN were inversely proportional in the NC, CB, and PC groups, but no such trend was observed in the NECB1 and NECB2 groups. Two-way analysis revealed that *C. butyricum* addition and NE infection at the same time affect the expression level of the protein OCLN, and the effect of interaction between *C. butyricum* and NE on OCLN expression was found to be significant (*p* < 0.01) (Figure 7B).

## Discussion

Necrotic enteritis in broilers is caused by *C. perfringens* types A, C, and G, which shows necrotic lesions in the intestinal mucosa of the broilers, thus reducing the FCR and causing severe economic losses in the broiler industry. *C. butyricum*, as a probiotic, can maintain the growth performance of broilers (Leeson et al., 2005). The supplement of *C. butyricum* in feed can promote growth performance and increase feed utilization (Yang et al., 2012; Zhang et al., 2014), improve intestinal barrier functions, and play an antagonistic effect against *C. perfringens* (Yang et al., 2012), which will mitigate the lesion score of NE and ameliorate the decreased ADG and increased FCR in NE (Takahashi et al., 2018). Similarly, our results also suggested that *C. butyricum* supplementation could improve the ADG and FCR in NE models caused by *C. perfringens* under the predisposing *E. maxima*.

The intestinal tissue morphology, villus height, and crypt depth are important indicators for the measurement of intestinal health, injury, and recovery (Golder et al., 2011). Furthermore, the nutrient absorption in the intestine potentized with the increase of intestinal epithelial transport and the height of the villus (Ruhnke et al., 2015). Dietary supplementation with probiotics such as *Lactobacillus acidophilus* and *C. butyricum* enhanced the restoration of intestinal morphology due to the increase of villus height, and the decrease of crypt depth (Zhang et al., 2016; Chen et al., 2018; Li et al., 2018). In this study, the NE broilers showed severe hemorrhage in the small intestinal tissue, the proliferation of inflammatory cells, shortening of the villus, and an increase of crypt depth. By comparison, the selected indices such as small intestinal lesion and crypt depth were improved in the broilers fed with *C. butyricum*, suggesting that *C. butyricum* was beneficial to NE broilers to maintain intestinal function.

The concentration of LPS in serum was used to evaluate the intestinal permeability (Shini et al., 2008; Gilani et al., 2016), and it is an indicator for evaluating the integrity and function of the intestinal barrier (Li et al., 2018). Our results showed that the serum LPS was increased in NE broilers but was not increased in NE broilers fed with *C. butyricum-*added diet, suggesting that *C. butyricum* aided in the maintenance of the intestinal function in NE broilers. These results are consistent with previous reports (Zhang et al., 2016; Li et al., 2018).

The *E. maxima* oocyst shedding result was as described in other studies, which showed that the addition of acid preparations or probiotics can improve coccidiosis, but the inhibition of oocysts of *Eimeria* spp. was not completely (Giannenas et al., 2012; Tonda et al., 2018). *C. butyricum* maybe improve intestinal microbial and pH to decrease oocyst shedding, without directly affect *E. maxima*. Therefore, in this experiment, we thought there was still have an amount of *E. maxima* that can be used as an inducement to cause chicken NE.

Tight junction proteins are vital for the integrity and function of the epithelial barrier. They maintain the diffusion barrier and seal the intercellular space. OCLN is a TJ protein that acts as an adhesion molecule between cells to maintain and regulate the barrier function of TJs. CLDNs also play an essential role in regulating cellular signal transduction and paracellular transport in the intestinal epithelium (Krause et al., 2008). ZO proteins are located on the surface of the inner cytoplasmic membrane. ZO-1 is crucial because it connects TJ proteins and the actin backbone (Furuse et al., 1994). Alpha-toxin of *C. perfringens* can damage the intestinal mucosal barrier (Rehman et al., 2006), and the expression of TJ proteins such as CLDN-1, CLDN-3, OCLN, and ZO-1 after NE decreased to varying degrees (Song et al., 2017; Zhang et al., 2017; Wu et al., 2019). NE leads to the increase in mRNA expression in the pore-forming TJ protein CLDN-2 (Zhang et al., 2017). Similar to CLDN-2, CLDN-5 is a pore-forming protein. In the present study, we observed that CLDN-5 increased with *C. perfringens*-induced NE but with supplementation of *C. butyricum*, the mRNA expression of CLDN-5 decreased. This showed that the addition of *C. butyricum* removed the decreasing tendencies of CLDN-1, CLDN-3, OCLN, and ZO-1 expression because of NE, thus confirming the results of previous studies (Song et al., 2017; Huang et al., 2019). MUCs interact with IgA and various growth factors to maintain a relatively stable intestinal environment (Fukuda, 2002). MUCs have potential binding sites for pathogenic microorganisms, and colonization of some bacteria could induce change in the expression of MUCs (Van Klinken et al., 1995). Furthermore, MUCs could provide nutrition for the proliferation of *C. perfringens* (Deplancke et al., 2002). After broilers develop NE, the expression of MUCs such as MUC2 and MUC5AC changed (Forder et al., 2012; Wu et al., 2018). Butyric acid could increase the mRNA expression of MUC2 inhibited by NE (Song et al., 2017). Our data are in agreement with these results.

Although *C. perfringens* may be present in the normal flora of healthy broilers, it is generally believed that a rapid proliferation of *C. perfringens* caused by some predisposing factors such as coccidiasis/coccidiosis (Opengart and Boulianne, 2019) will lead to NE in chickens. In healthy birds, *C. perfringens* is present in the range of 10^2^-10^4^ CFU/g intestinal contents, but in NE, the concentration of *C. perfringens* increased to 10^7^-10^9^ CFU/g (Shojadoost et al., 2012). In our results, the *C. perfringens plc* increased due to NE, but the supplementation of probiotics could affect the increase in *plc* level. The possible reason is that *C. butyricum* exerts a competitive exclusion effect on *C. perfringens*, thus reducing the growth of *C. perfringens* (Takahashi et al., 2018). Otherwise, bacteriocin (Nakanishi and Tanaka, 2010) and short-chain fatty acids (Antonissen et al., 2016; Xu et al., 2018; Huang et al., 2019) produced by *C. butyricum* may also help to reduce the risk of *C. perfringens* overgrowth. This finding indicates that *C. butyricum* can reduce the proliferation rate of *C. perfringens* and the intestinal barrier damage caused by NE.

Cytokines regulate cell growth and immune response and participate in the inflammatory response. Among these cytokines, TGFB1 is related to mucosal immune tolerance. IL-10 is mainly secreted by T cells and is an important anti-inflammatory cytokine, and IL-10 has a barrier protective effect (Scumpia and Moldawer, 2005). IL-6 is an effective pro-inflammatory cytokine of Th1 cells, mainly secreted by the intestinal epithelial cells (Al-Sadi, 2009). At the instance of NE, we observed that the expression of anti-inflammatory factors TGFB1, IL-10, and pro-inflammatory cytokines IL-6 increased. However, supplementation of *C. butyricum* lowered the upregulation of TGFB1, IL-10, and IL-6 observed in NE infection. Our findings support the increase in cytokine secretion after adding probiotics or food additives that can reduce inflammation after pathogen infection in broilers and pigs (Chen et al., 2018; Cao et al., 2019; Fasina and Lillehoj, 2019; Zhang et al., 2019). This result shows that an increase of immune factors caused by NE owing to *C. perfringens* stimulates the inflammatory immune response in the intestine. Notably, although no significant differences in serum IgA levels between NC and testing groups were observed, a similar downtrend of serum IgA in *C. butyricum* addition for the groups infected with NE (NECB1 and NECB2) has been shown, which was also reported in Zhang et al.'s (Zhang et al., 2014) results and the case of *C. butyricum* for *Escherichia coli K88* infection.

The Using chamber is a helpful tool for detecting the changes in the current and resistance of epithelial tissue by using electrodes to assess the integrity of the intestinal epithelial barrier. Evidence has been presented that after supplementing probiotic *Yeast Saccharomyces boulardii* in pig for 8 days, Gt remained unchanged, while Isc decreased, but Isc recovered after 16 days (Schroeder et al., 2004). Gt (tissue conductance) is a sign of tissue integrity, while Isc (short-circuit current) is a sign of net ion migration activity in the intestine, and the decrease of Gt and Isc indicates the closure of ion channels (Ruhnke et al., 2015). Previous work had reported that after broilers were infected with *Salmonella* or *Campylobacter*, Gt decreased because of the closure of ion channels, and the decrease of Gt was related to the decrease of net charge transfer of epithelial cells and the decrease in Isc (Awad et al., 2012, 2014). In the present study, Ussing chamber analysis showed that Gt was decreased in NE broilers, suggesting that the integrity of the intestinal epithelial barrier declined. Interestingly, the supplementation of *C. butyricum* could reverse the decrease in the values of Gt, signifying that *C. butyricum* can potentially maintain the integrity of the intestinal epithelial barrier in NE broilers. These results were in accordance with the expression patterns of CLDN-1, MUC-2, OCLN, and ZO-1 as indicated by the real-time PCR assay. However, some argue that the addition of organic acids did not affect the intestinal barrier (Ruhnke et al., 2015). The differential values of Gt and Isc in this study may be caused by the different dosages of *C. butyricum*. The FD4 flux between the intestinal epithelia mainly occurred through the cell bypass pathway. The increased flux of FD4 reflects the increased paracellular permeability and impaired intestinal barrier, which is inversely proportional to the OCLN expression level (Cani et al., 2009; Hu et al., 2013; Zhang et al., 2017). In the present study, when the *C. perfringens* infection-induced NE, the flux of FD4 increased. In contrast, the expression of OCLN decreased, indicating that the permeability of the intestine was increased, that is, the intestinal barrier was damaged. The addition of food additives such as L-arginine can inhibit the increase of FD4 flux caused by NE (Zhang et al., 2017). In the present study, the addition of *C. butyricum* showed the same result, indicating that *C. butyricum* can promote and maintain intestinal permeability of broilers.

## Conclusions

Our results showed that supplementing *C. butyricum* in the broiler chicken diet would be beneficial to improve the production performance of broilers under the NE caused by *C. perfringens*, in which *C. butyricum* reduced the colonization of *C. perfringens* and ameliorated the intestinal barrier function and integrity damaged by *C. perfringens*. In conclusion, *C. butyricum* can enhance broiler intestinal health and serve as an effective probiotic in poultry farming.

## Data Availability Statement

The raw data supporting the conclusions of this article will be made available by the authors, without undue reservation.

## Ethics Statement

The animal study was reviewed and approved by State Key Laboratory of Veterinary Etiological Biology, Lanzhou Veterinary Research Institute, Chinese Academy of Agricultural Sciences, China.

## Author Contributions

JC and XX designed the experiments. XX performed the experiments. SY provided the Ussing chamber method, and ZG and ZQ provided *E. maxima* oocyst (Guangdong strain). JW, YZ, HW, LX, and KZ participated in the animal experiments. XX wrote the manuscript. SY and JO helped in draft revision, and JC and EZ revised and finalized the manuscript. All authors approved the final version of the manuscript.

## Funding

This work was supported by Key Technologies Research and Development Program (Key Technologies R&D Program) 2017YFD050040320 and the Innovative Special Project of Agricultural Science and Technology (Grant No. CAAS-ASTIP-2014-LVRI-09) for JC.

## Conflict of Interest

The authors declare that the research was conducted in the absence of any commercial or financial relationships that could be construed as a potential conflict of interest.

## Publisher's Note

All claims expressed in this article are solely those of the authors and do not necessarily represent those of their affiliated organizations, or those of the publisher, the editors and the reviewers. Any product that may be evaluated in this article, or claim that may be made by its manufacturer, is not guaranteed or endorsed by the publisher.

## Supplementary Material

Supplementary Figure 1 Effect of diet *C. butyricum* on the macroscopic and pathological section results. **(A)** NC. **(B)** PC. **(C)** NECB2.

Supplementary Figure 2 Effect of diet *C. butyricum* on the macroscopic and pathological section results. **(A)** *E. max*. **(B)** ECB. Red arrow: inflammatory cell infiltration; Blue arrow: eosinophils. Scale bar = 50 μm.

Supplementary Figure 3 Effect of diet *C. butyricum* on *E. maxima* oocyst output. Each result represents the mean ± SEM (*n* = 3).
