# Supplementary figures and images for: Clostridium butyricum Supplement Can Ameliorate the Intestinal Barrier Roles in Broiler Chickens Experimentally Infected With Clostridium perfringens
Source: Front Physiol. 2021 Sep 24;12:737481. doi: 10.3389/fphys.2021.737481 (PMC8499529; doi:10.3389/fphys.2021.737481)

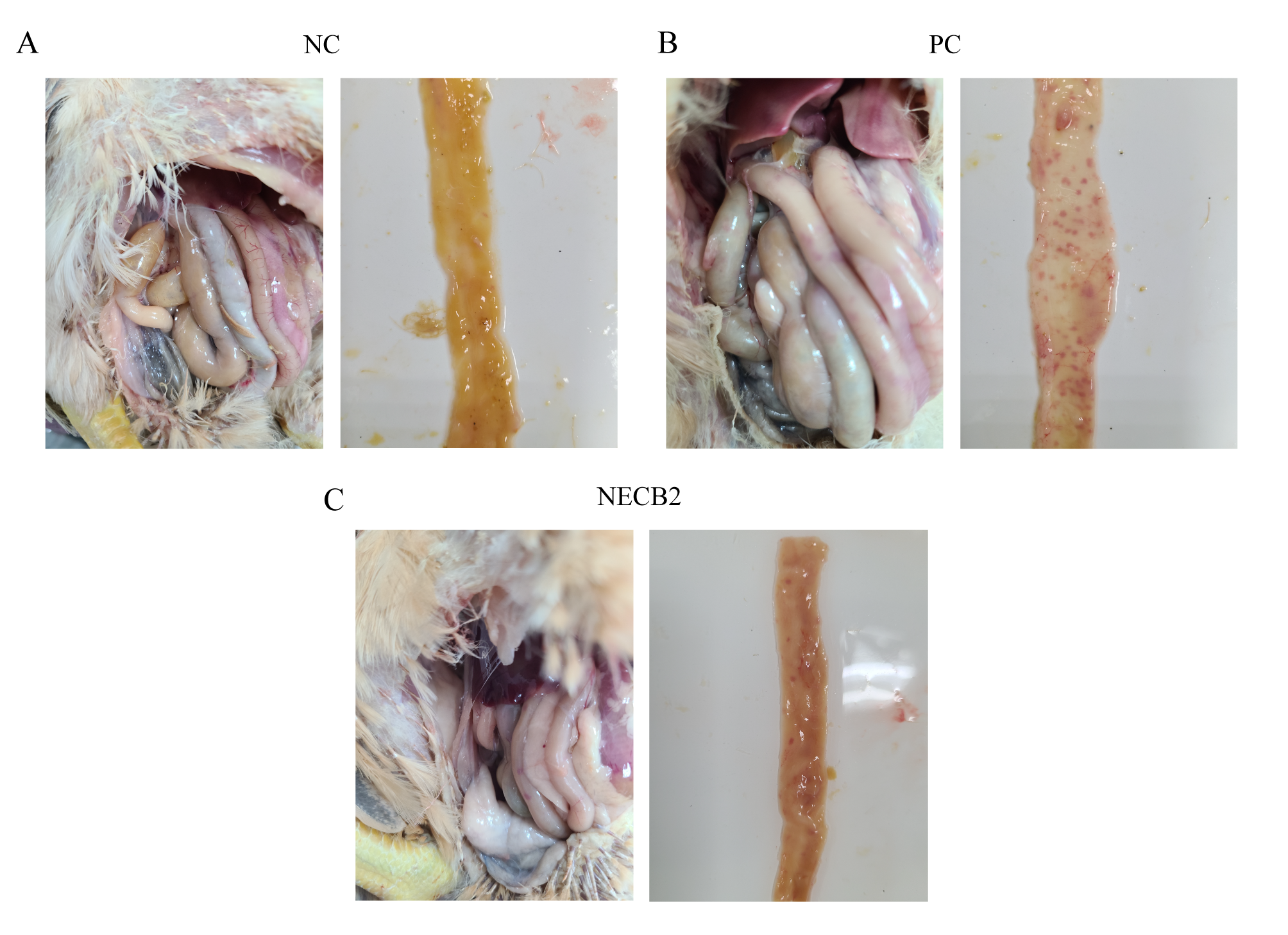

Supplement: Supplementary Figure 1 — Effect of diet C. butyricum on the macroscopic and pathological section results. (A) NC. (B) PC. (C) NECB2. [file Image_1.TIF]

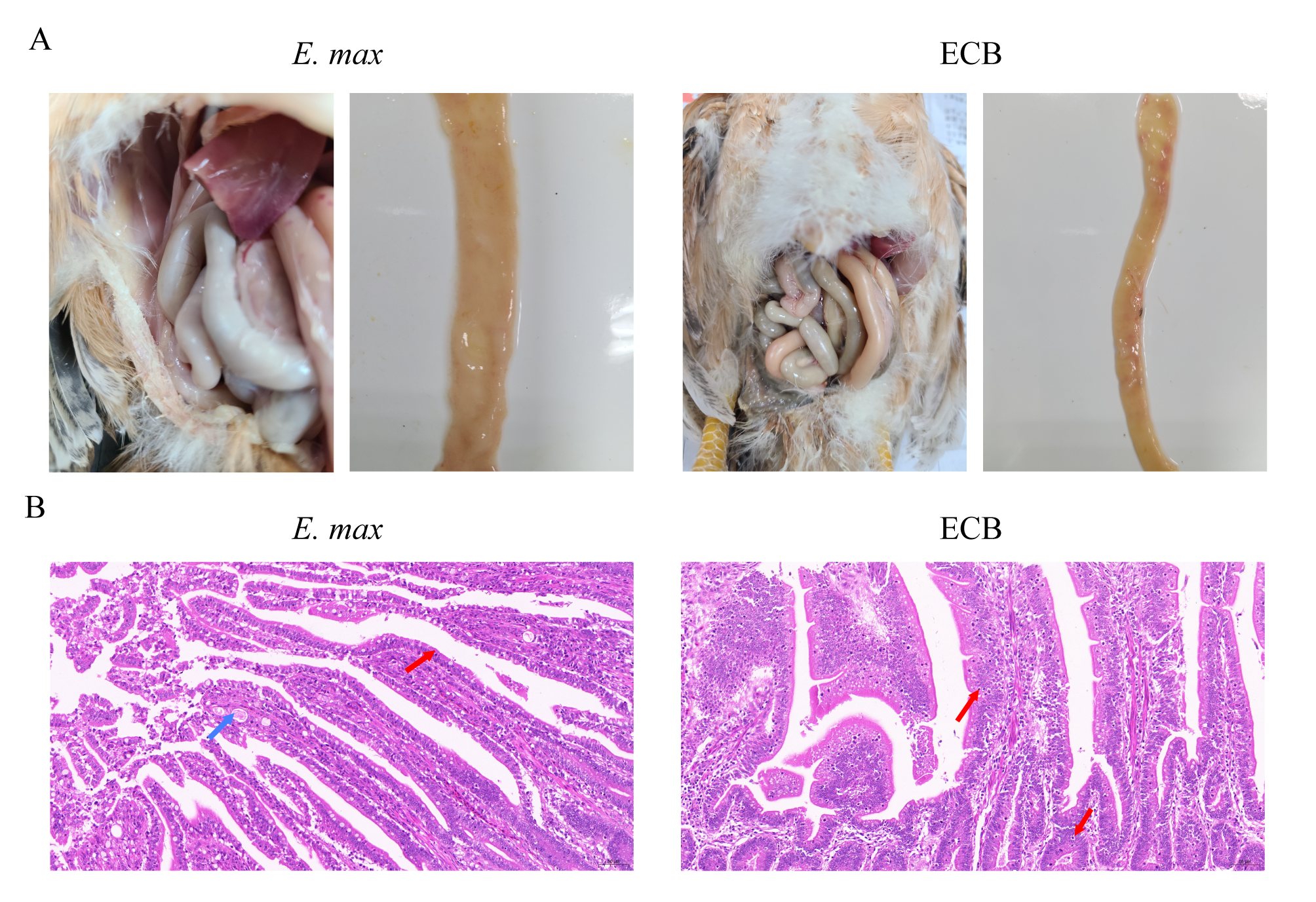

Supplement: Supplementary Figure 2 — Effect of diet C. butyricum on the macroscopic and pathological section results. (A) E. max. (B) ECB. Red arrow: inflammatory cell infiltration; Blue arrow: eosinophils. Scale bar = 50 μm. [file Image_2.TIF]

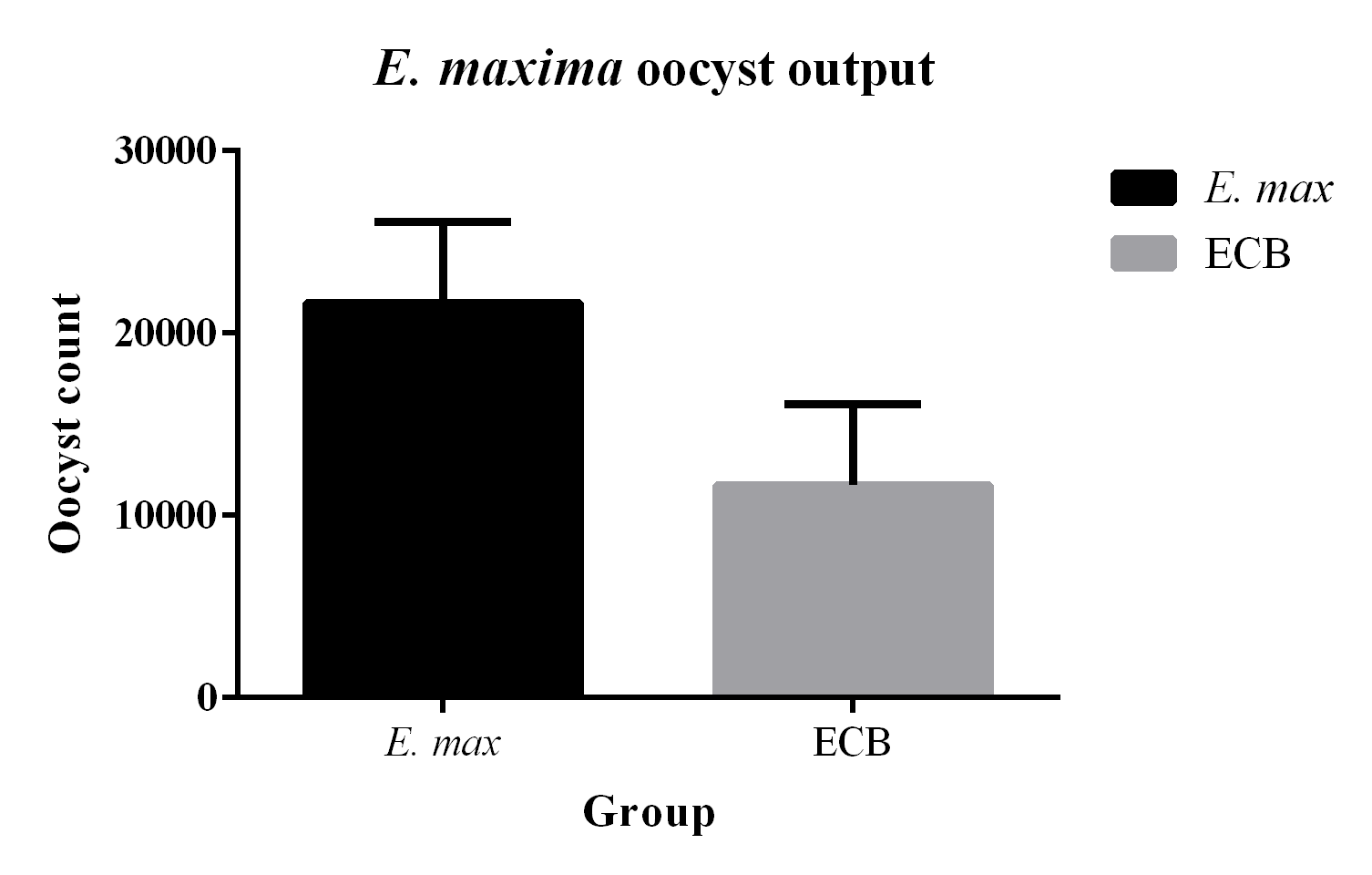

Supplement: Supplementary Figure 3 — Effect of diet C. butyricum on E. maxima oocyst output. Each result represents the mean ± SEM (n = 3). [file Image_3.TIF]
